# Climate Change Shapes Suitable Habitat and Ecological Niche Overlap Between *Hyphantria cunea* and Its Parasitoid *Chouioia cunea* in China

**DOI:** 10.1002/ece3.73469

**Published:** 2026-04-28

**Authors:** Xianheng Ouyang, Xiaoning Nan, Fushi Zhong, Qiaoyun Sun, Yang Liu

**Affiliations:** ^1^ College of Forestry Northwest A&F University Yangling Shaanxi China; ^2^ School of Architecture and Urban Planning Shenzhen University Shenzhen Guangdong China; ^3^ Key Laboratory of Resource Biology and Biotechnology in Western China (Ministry of Education) and College of Life Science Northwest University Xi'an Shaanxi Province China; ^4^ Department of Entomology University of Manitoba Winnipeg Manitoba Canada

**Keywords:** *Chouioia cunea*, ecological niche overlap, *Hyphantria cunea*, suitable habitat

## Abstract

The fall webworm moth, *Hyphantria cunea* , is a highly invasive defoliator that threatens forest ecosystems in China. The parasitoid wasp *Chouioia cunea* has been mass‐reared and widely released for biological control of *H. cunea* . Climate change can alter climatic suitability and potentially reshape the spatial matching between hosts and natural enemies, thereby affecting biological control outcomes. Here, we used an ensemble species distribution modeling (SDM) framework to project current (WorldClim 1971–2000) and future (2030s and 2050s) suitable habitats for *H. cunea* and *C. cunea* in China under three Shared Socioeconomic Pathways (SSP1‐2.6, SSP2‐4.5, and SSP5‐8.5). We quantified changes in suitable area and host‐parasitoid overlap and estimated climatic niche overlap using Schoener's *D*. Both species were projected to maintain broadly similar suitability patterns with a general northward shift and an increase in total suitable area under several scenarios, leading to extensive overlaps in eastern and central China. Schoener's *D* (0.738) indicated substantial climatic niche overlap between the two species. Among the retained predictors, the minimum temperature of the coldest month (bio6) and the Human Influence Index were most important for *C. cunea*. Under SSP5‐8.5, overlapping suitable areas were projected to increase to approximately 1.15 million km^2^ by the 2050s. These results provided a spatial basis for anticipating where biocontrol releases may be most effective and where potential host‐parasitoid mismatches could require strengthened monitoring under climate change.

## Graphical abstract

* *Hyphantria cunea* * is a highly invasive forest pest in China, and its parasitoid *Chouioia cunea* is widely used for biological control, though climate change may alter their spatial matching. Using ensemble species distribution models under multiple SSP scenarios, we found that suitable habitats for both species are projected to expand, with substantial niche overlap (Schoener's *D* = 0.738), particularly in eastern and central China. These results provide a basis for optimizing climate‐adaptive release strategies of *C. cunea* in future pest management programs.

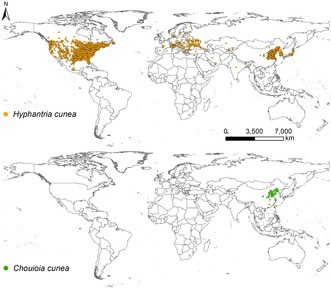

## 1 Introduction

With the acceleration of globalization and international trade, biological invasions have become increasingly frequent, posing global ecological and economic threats to ecosystem stability and native biodiversity (Pysek et al., Pyšek et al. 2020; Early et al. 2016). Invasive species disrupt the ecological balance through mechanisms such as resource competition, predation, and pathogen transmission, potentially leading to the decline or even extinction of native species and causing extensive and long‐lasting impacts on agriculture, forestry, and ecosystem services (Essl et al. 2020). Although traditional chemical control methods can effectively reduce pest populations in the short term, they often entail secondary risks such as environmental pollution, harm to non‐target species, and the development of pesticide resistance, making them unsustainable in the long run (Barathi et al. 2024). In contrast, biological pest control strategies, based on ecological regulatory mechanisms, are gaining recognition as key alternatives due to their high target species specificity and environmental compatibility (Van Lenteren et al. 2018). Biological control uses natural enemies to suppress pest populations, helping to limit pests to low densities while restoring and preserving the dynamic balance of ecosystems (Gurr et al. 2003). However, climate change is reshaping species' ecological niches and distribution patterns, potentially disrupting the spatiotemporal matching between pests and their natural enemies and thus posing new challenges to the effectiveness of biological control efforts (Ramos Aguila et al. 2023).

Species Distribution Models (SDMs), which integrate species occurrence records with environmental variables, are effective tools for inferring ecological niche characteristics and the distribution of suitable habitats. They play a critical role in assessing species invasion risks and formulating management strategies (Dubos et al. 2023). These models not only identify the potential distribution of species under current climatic conditions but can also predict their distribution dynamics under future climate scenarios. They have been widely used in studies involving the simulation of invasive species spread, optimization of natural enemy release areas, and the delineation of priority zones for pest control (Thomas et al. 2024). Common SDM modeling approaches include the Maximum Entropy Model (MaxEnt), Random Forest (RF), Gradient Boosting Machine (GBM), Generalized Linear Model (GLM), and Multivariate Adaptive Regression Splines (MARS), all of which can handle complex nonlinear relationships and high‐dimensional environmental factors, making them suitable for a variety of taxa and climatic contexts (Salako et al. 2023). The Biomod platform (https://sourceforge.net/projects/biomod2/) offers an ensemble modeling framework that integrates multiple algorithms to improve the stability and reliability of predictions and is especially valuable for ecological risk assessments under high‐uncertainty climate scenarios (Huang et al. 2024). Assessing ecological niche overlap between invasive species and their natural enemies can support the optimization of biological control strategies and address potential spatiotemporal mismatches under changing climatic conditions.

The fall webworm moth, *Hyphantria cunea* , is native to North America but has been introduced to parts of Europe and Asia, where it has become an important invasive defoliator (Nie et al. 2023). Since its introduction into China in the 1970s, it has spread rapidly across multiple provinces, causing severe damage to broadleaf trees such as poplar, willow, and elm, and posing a persistent threat to urban greenery and forest ecosystems (Ning et al. 2022). In China, the pupal parasitoid wasp *Chouioia cunea* is an indigenous natural enemy that has been mass‐reared and augmentatively released against the introduced *H. cunea* (Yang et al. 2006). Because *C. cunea* is native to East Asia, its natural distribution does not necessarily match the native range of *H. cunea* in North America, and its observed occurrences may also be influenced by release programmes. Spatial ecological niche mismatches between the pest and its parasitoid, together with climate‐driven shifts in suitability, could influence the regional effectiveness of biological control (Ramos Aguila et al. 2023). Therefore, assessing potential suitable habitats and climatic niche overlap of *H. cunea* and *C. cunea* under current and future climate conditions is important for anticipating where biological control may be effective and where additional monitoring may be required.

This study used species distribution models (SDMs) to evaluate patterns of climatic suitability and potential spatial overlap between *H. cunea* and its parasitoid *C. cunea* under current and projected future climate scenarios. We used three Shared Socioeconomic Pathways (SSP1‐2.6, SSP2‐4.5 and SSP5‐8.5) to project distributions for the 2030s and 2050s, quantify potential changes in suitable area (expansion, contraction and stability), and identify regions where host suitability may occur with limited parasitoid suitability. Our goal was to provide a spatially explicit basis for risk monitoring and for optimizing the release strategy of *C. cunea* under climate change.

## 2 Materials and Methods

### 2.1 Occurrence Records

**FIGURE 1:**
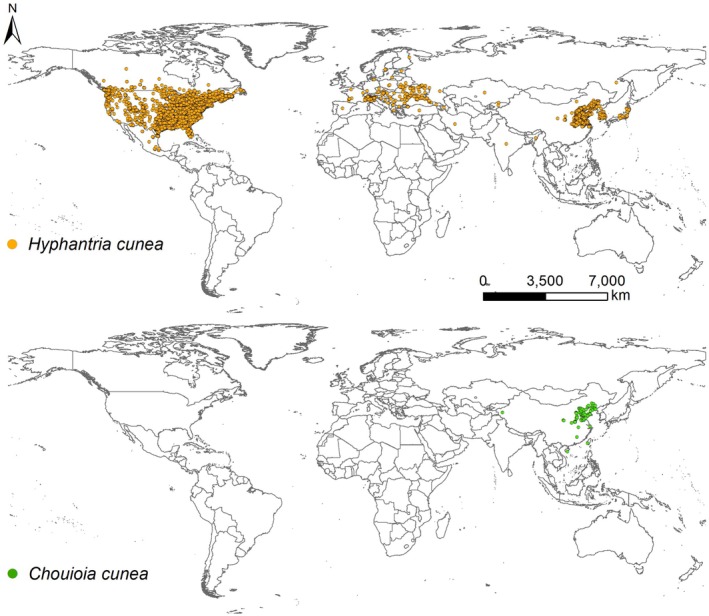
Current global distribution records of *Hyphantria cunea* and its parasitoid, *Chouioia cunea*, used for model calibration. *Hyphantria cunea* is native to North America and invasive in China, whereas *C. cunea* is an indigenous parasitoid in China that has been used for biological control of *H. cunea* .

In this study, occurrence records of *H. cunea* and its parasitoid *C. cunea* were compiled primarily from the Global Biodiversity Information Facility (GBIF, https://www.gbif.org) and the Barcode of Life Data System (BOLD, https://www.boldsystems.org). In addition, we screened peer‐reviewed papers and official monitoring reports that provided verifiable locality information (hereafter referred to as “relevant literature”) to supplement records and to verify doubtful occurrences. To reduce spatial sampling bias and spatial autocorrelation, we applied spatial thinning using ENMTools (http://enmtools.com/), retaining one occurrence per 5 × 5 km grid cell. After filtering, 10,737 records for *H. cunea* and 82 records for *C. cunea* were retained for modeling (Figure 1).

Species distribution modeling often assumes that occurrences approximate equilibrium with the environment (i.e., ecological niche saturation). Because both species are affected by invasion history and, for *C. cunea*, potential human‐mediated releases, this assumption may be imperfect. We therefore cross‐checked the cleaned occurrence dataset against the country‐level distributions reported in the Centre for Agriculture and Bioscience International Invasive Species Compendium (CABI‐ISC, https://www.cabidigitallibrary.org) to ensure that no major invaded regions were missing due to data access or filtering. We did not generate additional pseudo‐occurrences for unsampled areas; instead, remaining spatial gaps are treated as potential sampling bias and are discussed as a limitation. Models were calibrated using all cleaned occurrences and were projected and interpreted for China to support management‐oriented inference. The climate predictors used below represent long‐term means (WorldClim 1971–2000); therefore, we did not restrict occurrences to a single collection year, and we interpret the “current” suitability as the climatic baseline rather than a snapshot of a specific year.

### 2.2 Environmental Data

The environmental variables used in this study were obtained from the WorldClim 2.1 database (https://www.worldclim.org/data/worldclim21.html) and include 19 bioclimatic variables (bio1‐bio19) and elevation at 2.5 arc‐min resolution (Table S1). For the current climate, we used the 1971–2000 baseline climatology (30‐year means). Future climate projections for the 2030s (2021–2040) and 2050s (2041–2060) were downloaded from WorldClim based on the BCC‐CSM2‐MR global climate model under SSP1‐2.6, SSP2‐4.5 and SSP5‐8.5 (Wu et al. 2019) (Table S1). To account for anthropogenic effects potentially relevant to the managed distribution of *C. cunea*, we included the Human Influence Index (HII; 1995–2004), a composite index summarizing human population pressure, land use and accessibility, from NASA's Socioeconomic Data and Applications Center (SEDAC) at 30 arc‐second resolution (https://sedac.ciesin.columbia.edu/data/set/wildareas‐v2‐human‐influence‐index‐geographic). All predictors were resampled to a common 2.5 arc‐min grid in ArcGIS 10.8, and environmental values were extracted for each occurrence record. To reduce multicollinearity, we calculated pairwise Pearson correlations and removed one variable from each highly correlated pair (|r| > 0.8), retaining the predictor with clearer mechanistic relevance to the biology of the insects (e.g., we prioritized cold‐season temperature (bio6) because overwinter survival can be limiting, and dry‐season precipitation (bio14) because drought can constrain host plant condition). Eight predictors (six bioclimatic variables, elevation and HII) were retained for modeling (Table S2).

### 2.3 Model Construction

Potential suitable habitat modeling was performed in R (R Core Team 2024) using the BIOMOD2 package (Thuiller et al. 2009), comparing five algorithms: generalized boosting model (GBM; Friedman 2001), generalized linear model (GLM; McCullagh 2019), multivariate adaptive regression splines (MARS; Friedman 1991), random forest (RF; Breiman 2001) and maximum entropy (MaxEnt; Phillips et al. 2006). For each species, we randomly split occurrences into 75% training and 25% testing data, and generated 10,000 pseudo‐absence points within the modeling background (excluding grid cells containing occurrences) to improve model discrimination. Each algorithm was fitted with 10 replicate runs to account for stochasticity. Predictive performance was evaluated using the area under the receiver operating characteristic curve (AUC) and the true skill statistic (TSS) (Zhao et al. 2023). To construct the ensemble model, algorithms were ranked by mean performance (averaged across AUC and TSS), and the four best‐performing methods (RF, GBM, MARS and MaxEnt) were retained for both *H. cunea* and *C. cunea*. Ensemble predictions were obtained as a weighted average of the retained models (weights proportional to TSS) and were projected for the baseline climate as well as for the 2030s and 2050s under SSP1‐2.6, SSP2‐4.5 and SSP5‐8.5. Predictor importance was quantified using BIOMOD2's permutation‐based variable importance (unitless, 0–1), where higher values indicate stronger influence on model predictions.

### 2.4 Potential Area of *H. cunea* and *C. cunea* Geographic Distribution Overlap

For spatial overlap analyses, continuous ensemble suitability outputs were converted to binary suitable/unsuitable maps using the maximum training sensitivity plus specificity threshold (MTSS; Liu et al. 2005) in ArcGIS. For visualization in the maps, suitability values were rescaled to 0–1000; grid cells below the MTSS threshold were treated as unsuitable, whereas suitable cells (above MTSS) were further classified as low (MTSS‐400), moderate (400–600) and high (600–1000) suitability. Importantly, overlap was calculated using the binary suitability maps (i.e., all suitable classes combined) by overlaying the predicted suitable areas of the two species.

### 2.5 Measurement of Ecological Niche

We assessed climatic niche overlap and niche dynamics using the ecospat R package (Di Cola et al. 2017) following the COUE framework (Centroid shift, Overlap, Unfilling and Expansion) (Broennimann et al. 2012). For *H. cunea* , we compared the realized climatic niche between its native range and invaded range to evaluate niche equivalency and niche similarity. The bidirectional similarity test (native ‐ > invaded and invaded ‐ > native) evaluates whether the niche in one range is more similar to the other than expected under a randomization of occurrences within the available climatic background (1000 permutations). Schoener's *D* was used as an effect‐size measure of climatic niche overlap (0 = no overlap; 1 = complete overlap) for both the comparison between *H. cunea* and *C. cunea* and the comparison between native and invaded ranges. Because *p*‐values from permutation tests can be sensitive to sample size, we interpret them cautiously and focus primarily on the magnitude of Schoener's *D*.

## 3 Results

### 3.1 Model Accuracy Evaluation

**FIGURE 2:**
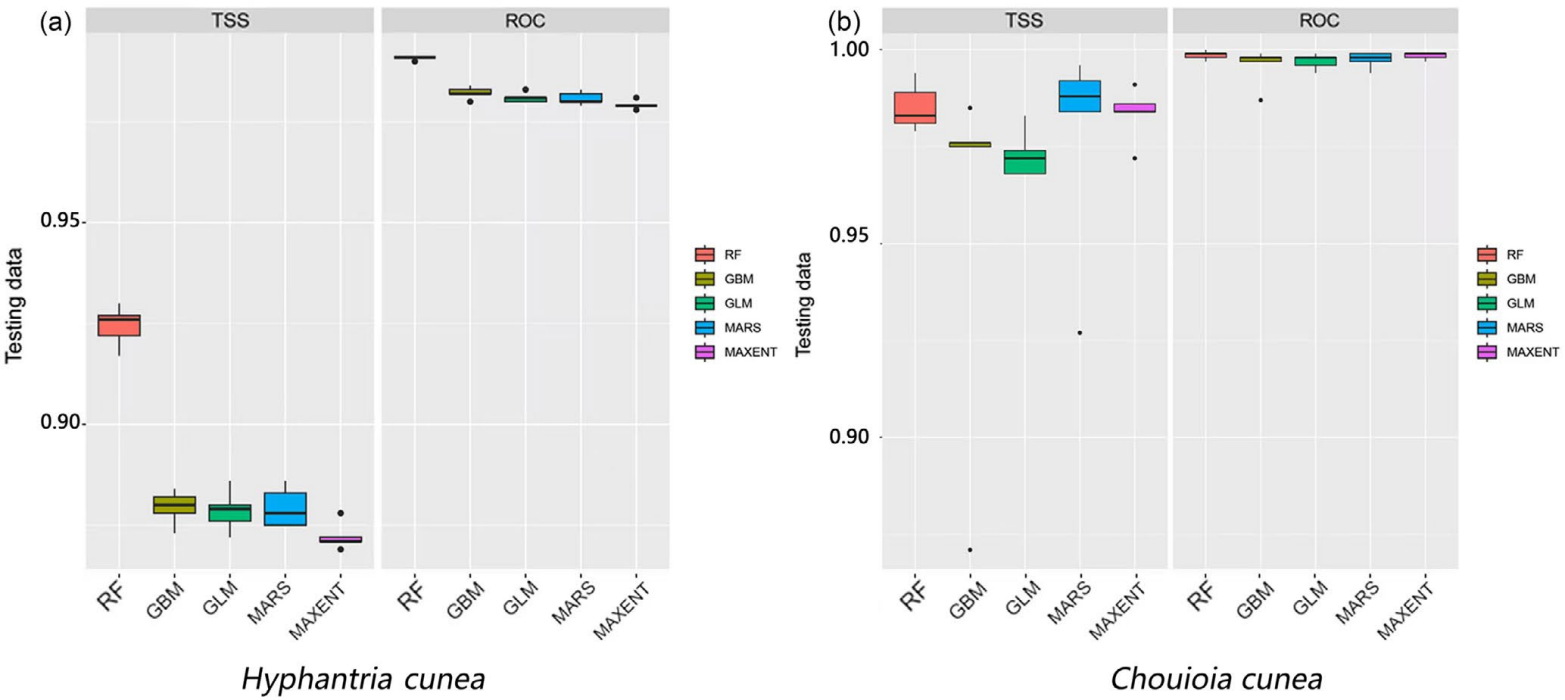
Model evaluation using (a) true skill statistic (TSS) and (b) area under the receiver operating characteristic curve (AUC) for five algorithms (RF, GBM, GLM, MARS and MaxEnt) predicting the potential distributions of *Hyphantria cunea* and *Chouioia cunea*. RF, random forest; GBM, generalized boosting model; GLM, generalized linear model; MARS, multivariate adaptive regression splines; MaxEnt, maximum entropy. The ensemble model built from the four best‐performing algorithms achieved mean TSS > 0.83 and mean AUC > 0.96.

We evaluated the performance of the five single‐species distribution model algorithms (RF, GBM, GLM, MARS, and MaxEnt) in predicting the potential distributions of *H. cunea* and its parasitoid *C. cunea* using TSS and AUC (Figure 2). Mean TSS values exceeded 0.80 and mean AUC values exceeded 0.95 for both species, indicating good model discrimination. Following the selection procedure described in the Methods, we retained the four best‐performing algorithms (RF, GBM, MARS, and MaxEnt) to build the ensemble model. The ensemble achieved mean TSS values above 0.83 and mean AUC values above 0.96, and was therefore used for all subsequent projections.

### 3.2 Current Suitable Habitat and Distribution of Habitat Overlaps Between *H. cunea* and *C. cunea*

**FIGURE 3:**
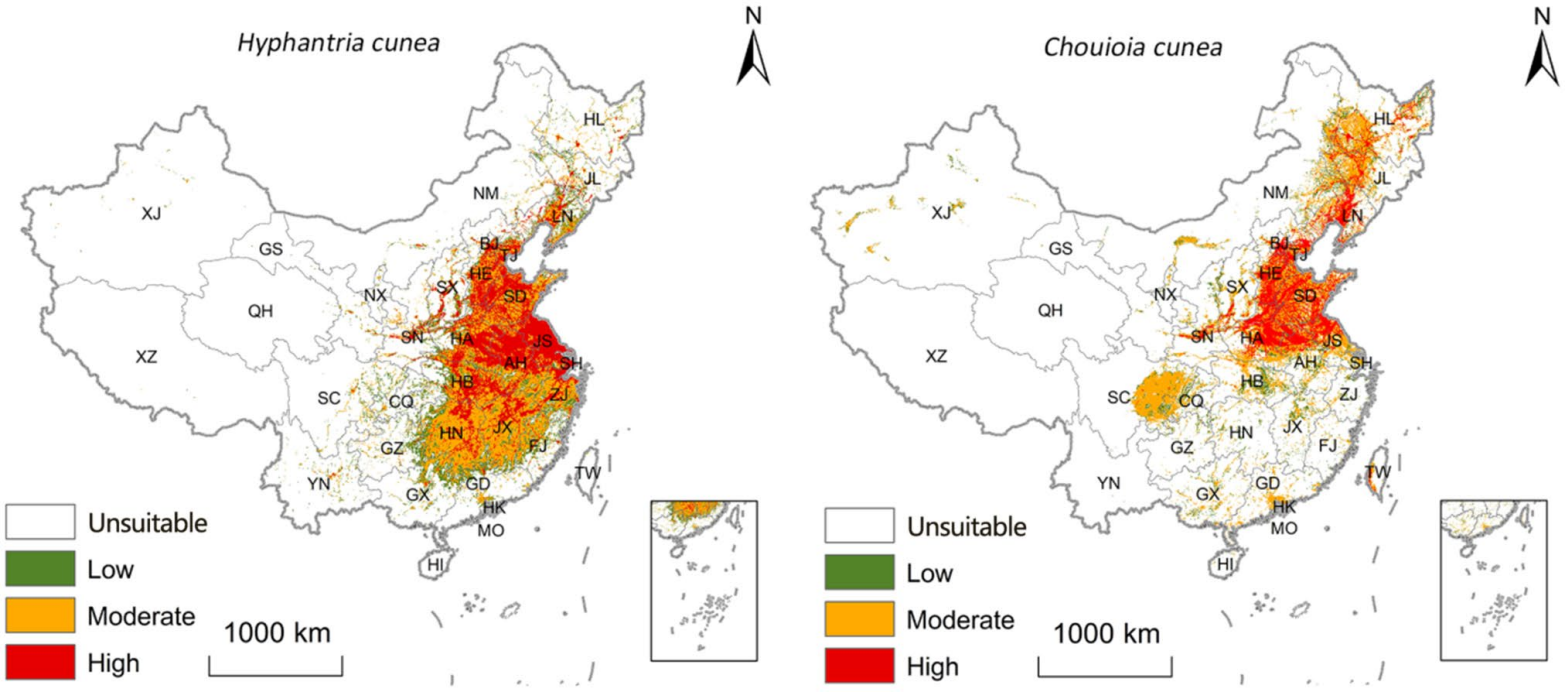
Current geographical distributions of *Hyphantria cunea* and its insect parasitoid, *Chouioia cunea*, in China, using the Ensemble Model.

Under current climate conditions, the total potential suitable habitat area for *H. cunea* was 1.63 million km^2^ (Table 1; Figure 3; areas in Table 1 are reported as ×10^4^ km^2^). Suitable habitats were widely distributed across Northeast, North, East, and Central China, and parts of Southwest China, including provinces such as Heilongjiang, Jilin, Liaoning, Beijing, Tianjin, Hebei, Shandong, Henan, Jiangsu, Hubei, and Sichuan. Highly suitable habitat accounted for 36.53% of the total (0.60 million km^2^) and was mainly concentrated in the North China Plain, the middle and lower reaches of the Yangtze River Basin, and the Sichuan Basin.

**TABLE 1:** Potential suitable areas for *Hyphantria cunea* and *Chouioia cunea* under different climate change conditions (10^4^km^2^).

Species	Period	Climate scenario	Area (×10^4^km^2^)			
			Poorly	Moderately	Highly	Total
*Hyphantria cunea*	current	—	27.94	75.82	59.73	163.49
	2030s	SSP1‐2.6	33.81	85.86	50.62	170.29
		SSP2‐4.5	35.08	78.98	52.19	166.25
		SSP5‐8.5	34.86	76.26	40.71	151.83
	2050s	SSP1‐2.6	31.16	87.04	58.49	176.69
		SSP2‐4.5	38.41	85.74	42.57	166.73
		SSP5‐8.5	41.33	100.37	49.43	191.14
*Chouioia cunea*	Current	—	17.30	77.52	38.67	133.49
	2030s	SSP1‐2.6	24.68	95.06	38.08	157.82
		SSP2‐4.5	23.85	98.79	42.38	165.02
		SSP5‐8.5	22.88	100.86	36.86	160.60
	2050s	SSP1‐2.6	23.67	89.72	34.33	147.72
		SSP2‐4.5	30.17	102.97	34.53	167.67
		SSP5‐8.5	31.34	104.68	38.88	174.89

Suitable habitat for *C. cunea* was mainly distributed in East, Central and South China, including Jiangsu, Zhejiang, Jiangxi, Hunan, Guangdong, and Guangxi provinces (Table 1; Figure 3). The total suitable area for *C. cunea* was 1.33 million km^2^, with highly suitable areas accounting for 28.97% (0.39 million km^2^).

**FIGURE 4:**
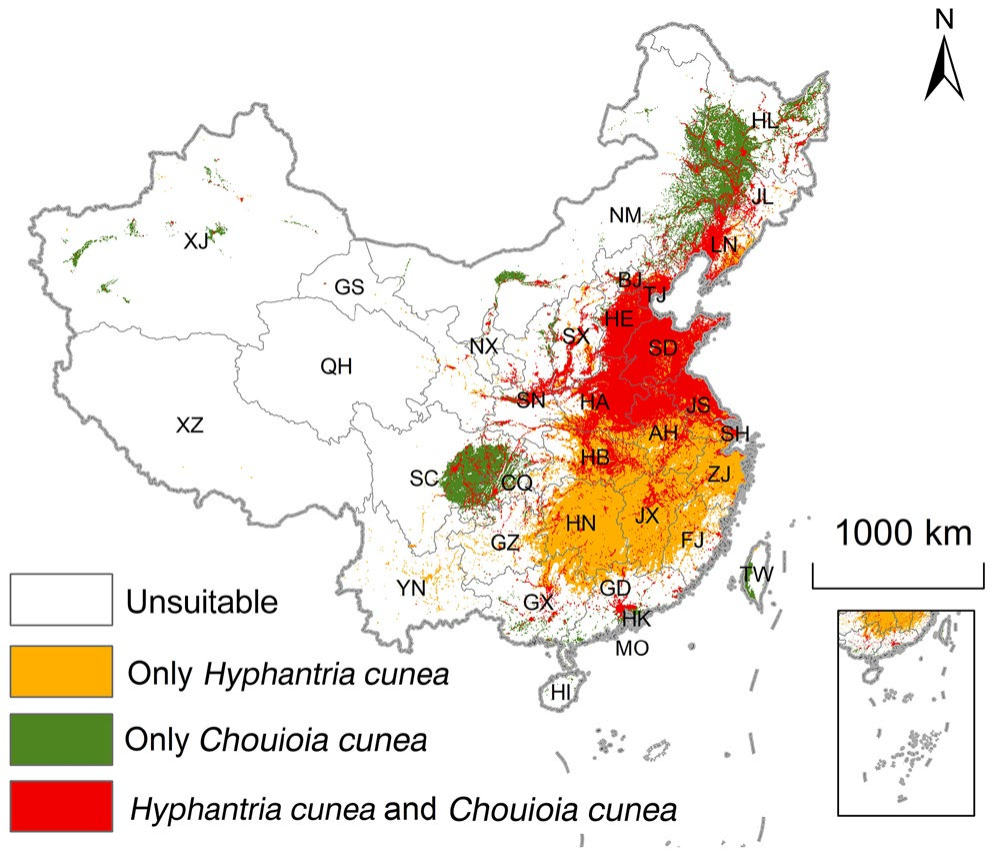
Overlapping geographical distribution areas of *Hyphantria cunea* and its parasitoid *Chouioia cunea* in China, based on binary suitable areas (all grid cells above the MTSS threshold; low, moderate, and high suitability classes combined).

The geographic overlap area between the two species was 0.89 million km^2^, accounting for 54.46% of the suitable area for *H. cunea* (Table 2; Figure 4). This overlapping distribution was mainly concentrated in southern North China, East China (e.g., Jiangsu and Anhui), Central China, and eastern Southwest China (e.g., Hubei and Chongqing), indicating substantial co‐suitability between the parasitoid and its host under the current climate baseline.

**TABLE 2:** Overlapping area of globally projected geographical distribution of *Hyphantria cunea* and *Chouioia cunea* under climate conditions. A: *Hyphantria cunea.* B: *Chouioia cunea*.

Period	Scenario	Overlapping area (×10^4^km^2^)		
		A ( *H. cunea* ) only (×10^4^ km^2^)	B (C. cunea) only (×10^4^ km^2^)	A and B
current	—	74.45	44.45	89.04
2030s	SSP1‐2.6	71.14	58.67	99.15
	SSP2‐4.5	63.28	62.05	102.97
	SSP5‐8.5	54.13	62.91	97.70
2050s	SSP1‐2.6	83.64	54.67	93.05
	SSP2‐4.5	62.63	63.57	104.10
	SSP5‐8.5	76.11	59.86	115.03

### 3.3 Changes in Suitable Habitat Under Future Climate Scenarios

**FIGURE 5:**
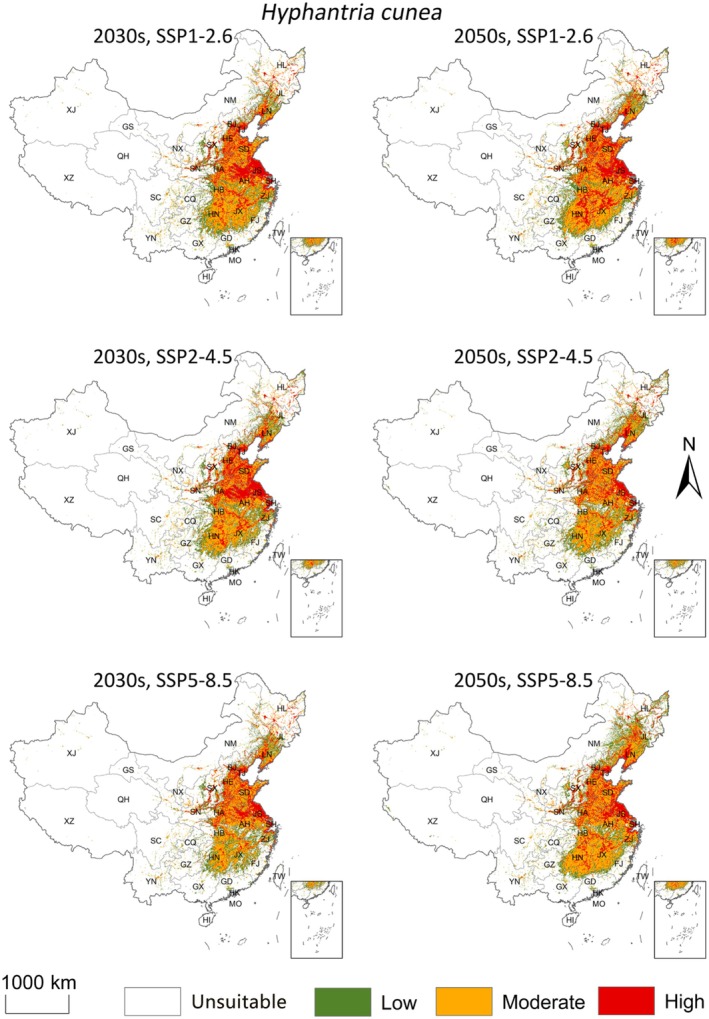
Ensemble Model‐based simulation of the potential suitable areas for *Hyphantria cunea* in China under postulated future climate conditions. SSP1‐2.6, SSP2‐4.5, and SSP5‐8.5: Low, medium, and high emission scenarios, respectively.

The distributions of suitable habitat for *H. cunea* varied across SSP1‐2.6, SSP2‐4.5, and SSP5‐8.5 scenarios (Table 1; Figure 5).

In the 2030s, under SSP1‐2.6, the suitable area for *H. cunea* expanded northeastward, with new suitable regions emerging in northern Heilongjiang and eastern Jilin, increasing the total suitable area to 1.70 million km^2^. Under SSP5‐8.5, suitable habitats in southern provinces (e.g., Guangdong and Guangxi) decreased, and the total suitable area declined to 1.52 million km^2^ (Table 1; Figure 5).

In the 2050s, under SSP5‐8.5, the suitable area for *H. cunea* increased to 1.91 million km^2^, with expansions occurring in the northeast (e.g., Heilongjiang and Jilin) and northwest (e.g., northern Xinjiang) (Table 1; Figure 5).

**FIGURE 6:**
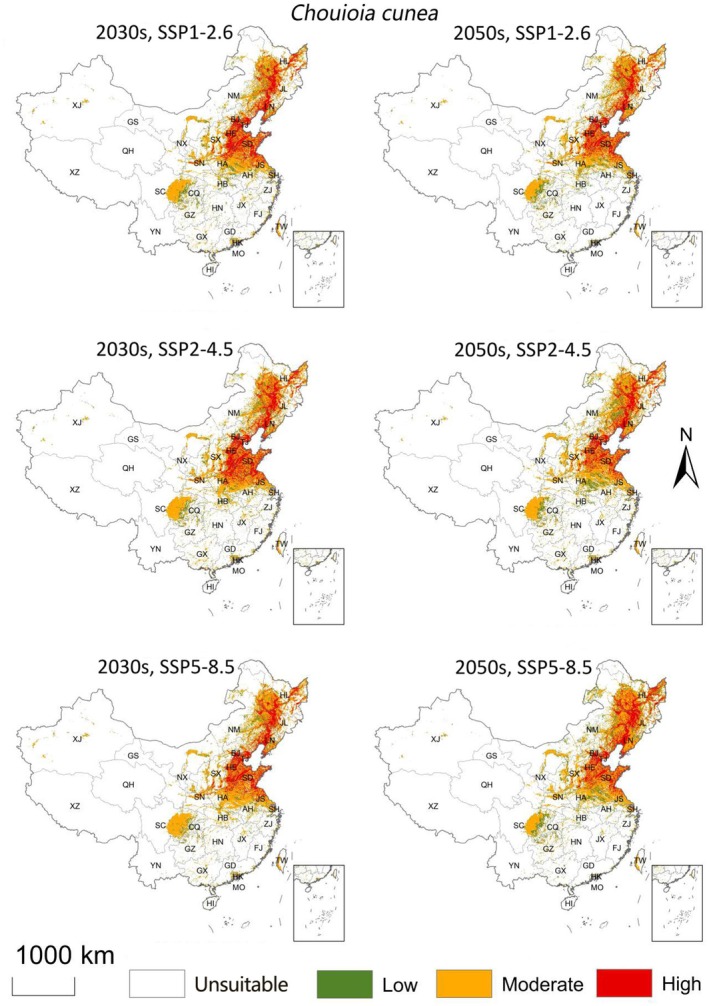
Ensemble Model‐based simulation of the potential suitable areas for *Chouioia cunea* in China under postulated future climate conditions. SSP1‐2.6, SSP2‐4.5, and SSP5‐8.5: Low, medium, and high emission scenarios, respectively.

Predicted suitability for *C. cunea* also changed across scenarios, but the overall spatial pattern remained concentrated in eastern and central China (Table 1; Figure 6).

In the 2030s, under SSP2‐4.5, the total suitable area for *C. cunea* reached its maximum (1.65 million km^2^), with newly suitable regions appearing in North China, southern Northeast China (e.g., Liaoning), and parts of Northwest China (e.g., Shaanxi). Under SSP5‐8.5, the area of highly suitable habitat decreased and became more concentrated along the Yangtze River Basin and the North China Plain (Table 1; Figure 6). In the 2050s, under SSP5‐8.5, the total suitable habitat increased to 1.75 million km^2^, with northern and high‐altitude regions such as Inner Mongolia and eastern Qinghai becoming newly suitable (Table 1; Figure 6).

### 3.4 Overlapping Areas and Dynamic Changes

**FIGURE 7:**
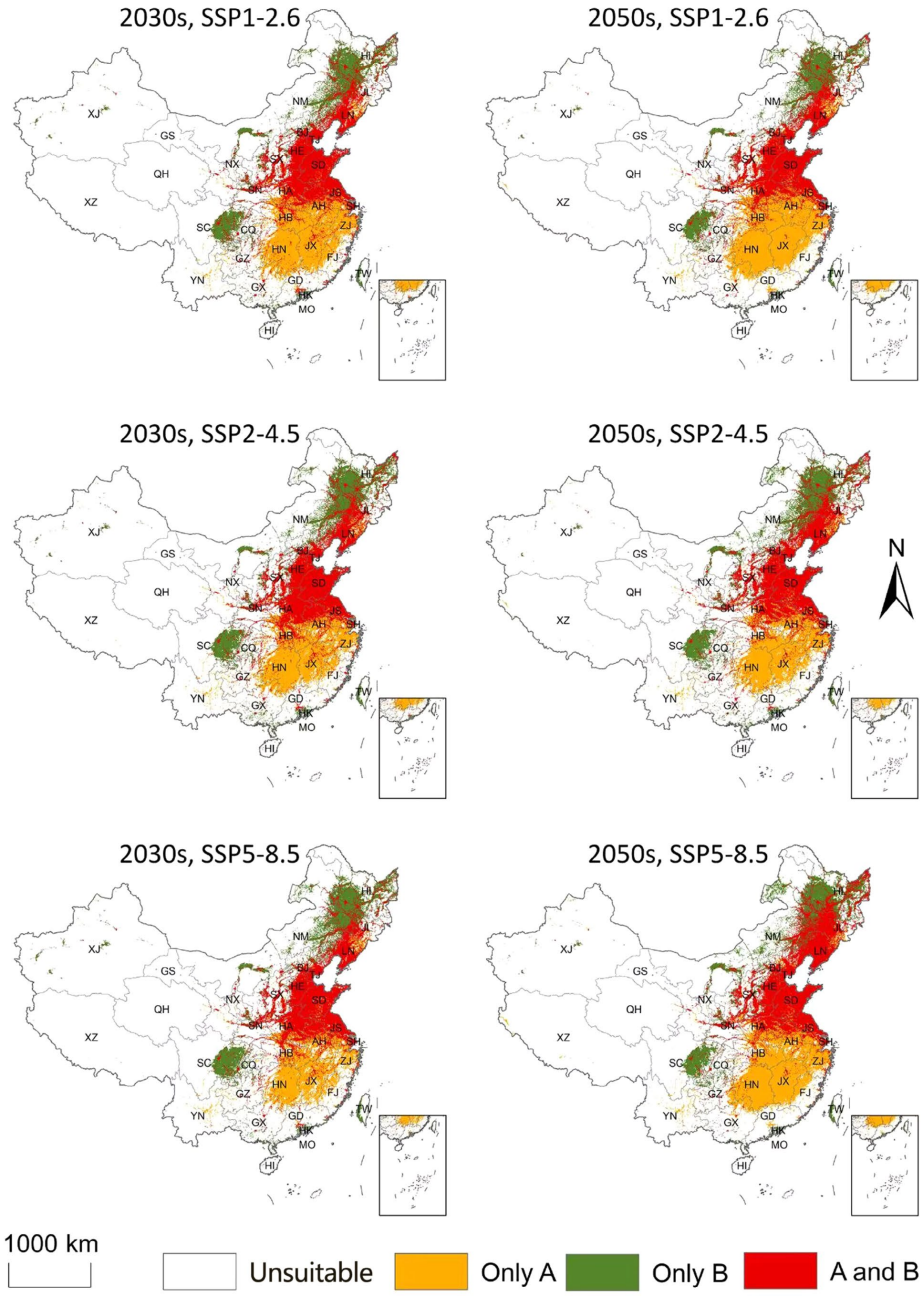
Projected overlapping areas of distribution of *Hyphantria cunea* and its parasitoid *Chouioia cunea* in China under different future climate scenarios. A, *Hyphantria cunea* ; B, *Chouioia cunea*; SSP1‐2.6, SSP2‐4.5, and SSP5‐8.5: Low, medium, and high emission scenarios, respectively.

Tables 2, 3 and S3 and Figures 7, S1 and S2 summarize the dynamic changes in the overlapping suitable areas of *H. cunea* and the parasitoid *C. cunea* under different future climate scenarios. Under the current climate baseline, the overlapping suitable area was 0.89 million km^2^, accounting for 54.46% of the suitable habitat for *H. cunea* , and was mainly concentrated in southern North China, East China, Central China, and eastern Southwest China (Table 2; Figure 4). In the 2030s, overlap increased to 0.99 million km^2^ under SSP1‐2.6 and 1.03 million km^2^ under SSP2‐4.5, reflecting a northward expansion of co‐suitable habitat, whereas under SSP5‐8.5 overlap was 0.98 million km^2^ with reduced co‐suitability in some southern areas (Table 2; Figure 7). In the 2050s, overlap was projected to be 0.93 million km^2^ (SSP1‐2.6), 1.04 million km^2^ (SSP2‐4.5) and 1.15 million km^2^ (SSP5‐8.5), with the largest overlap under SSP5‐8.5 extending to northern and higher‐elevation regions (Table 2; Figure 7). Dynamic change analysis indicated that overlap expansion and stability dominated across scenarios, with relatively little contraction, particularly under SSP5‐8.5 (Table 3; Figure S2).

**TABLE 3:** Changes in the overlapping area of potential geographical distribution (×10^4^ km^2^) for *Hyphantria cunea* and *Chouioia cunea* under future climate scenarios.

Period	Scenario	Degree of habitat suitability (×10^4^ km^2^)		
		Contraction	Stability	Expansion
2030s	SSP1‐2.6	7.77	81.28	17.88
	SSP2‐4.5	6.29	82.76	20.21
	SSP5‐8.5	8.21	80.83	16.86
2050s	SSP1‐2.6	15.27	73.77	19.28
	SSP2‐4.5	9.82	93.15	10.95
	SSP5‐8.5	9.36	88.34	26.70

### 3.5 Impact of Environment Variables on the Potential Geographical Distributions of *H. cunea* and *C*. *Cunea*

Based on the ensemble‐model variable importance in biomod2 (Table 4, values scaled from 0 to 1, where higher values indicate higher importance), precipitation of the driest month (bio14, 0.18), minimum temperature of the coldest month (bio6, 0.16) and the human influence index (HII, 0.11) were the three highest‐ranked predictors for *H. cunea*.

**TABLE 4:** Contribution of each variable for *Hyphantria cunea* and *Chouioia cunea* based on EM.

*Hyphantria cunea*		*Chouioia cunea*	
Environmental variable	Contribution (%)	Environmental variable	Contribution (%)
altitude	0.00	altitude	0.01
bio12	0.04	bio12	0.02
bio14	0.18	bio14	0.01
bio15	0.01	bio15	0.39
bio2	0.00	bio2	0.17
bio5	0.09	bio5	0.30
bio6	0.16	bio6	0.46
hii	0.11	hii	0.52

For *C. cunea*, HII had the highest importance (0.52), followed by bio6 (0.46) and precipitation seasonality (bio15, 0.39), suggesting that both climatic constraints and anthropogenic factors contributed to the predicted distribution of *C. cunea* (Table 4).

### 3.6 Ecological Niche Overlap and Similarity

**FIGURE 8:**
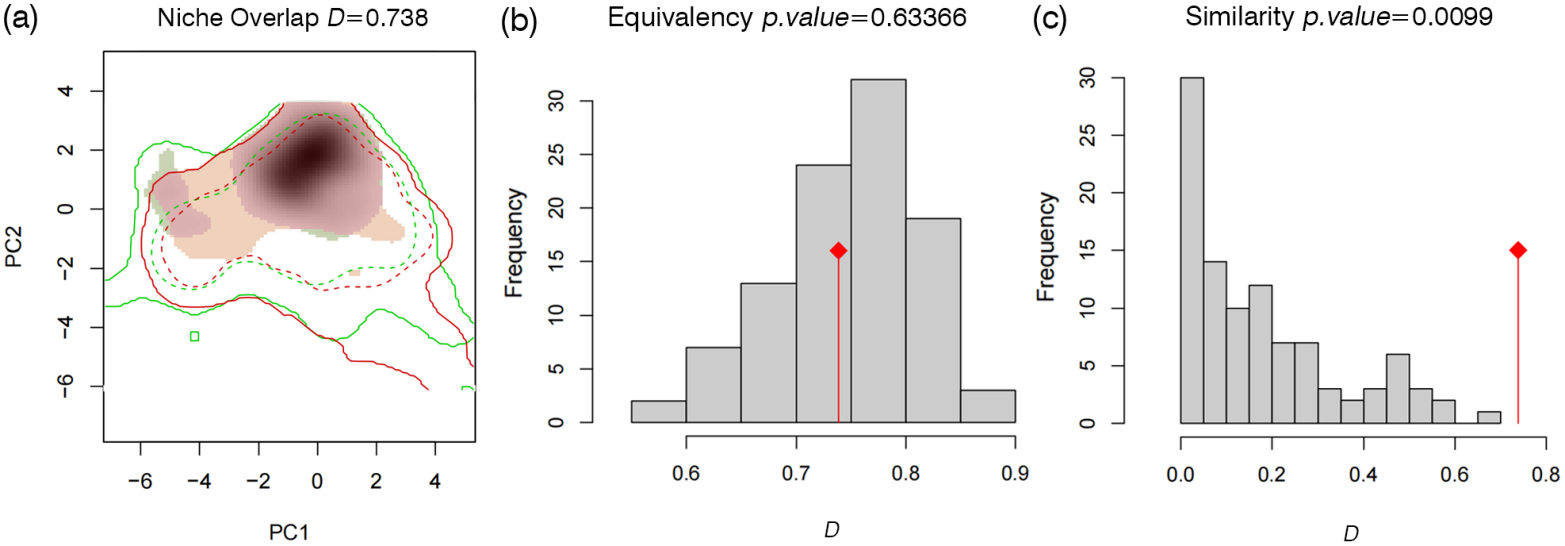
Ecological niches of *Hyphantria cunea* and *Chouioia cunea* in climatic space derived from a principal component analysis (PCA) of eight predictors, and niche tests for *H. cunea* between its native and invaded ranges. (a) Niche overlap index (Schoener's *D* = 0.738) indicates substantial climatic niche overlap between the two species; green represents *Chouioia cunea* (parasitoid wasp), yellow represents *Hyphantria cunea* (fall webworm moth), and red indicates the overlapping area. (b) Equivalency test (*p* = 0.63366) does not reject the null hypothesis of “niche equivalency” between the native and invaded ranges of *H. cunea* . (c) Similarity test (*p* = 0.0099) rejects the null hypothesis of “niches are not similar” (native vs. invaded).

Based on Schoener's *D*, climatic niche overlap between *H. cunea* and *C. cunea* was substantial (*D* = 0.738; Figure 8a), indicating that the two species occupy broadly similar portions of the climatic space defined by the eight predictors. In the PCA climate space (PC1 and PC2 represent the first two principal components of the eight predictors), the niches of the two species showed considerable overlap and their centroid positions were close. We also assessed niche equivalency and similarity for *H. cunea* between its native and invaded ranges (Figure 8b,c); these permutation tests should be interpreted cautiously given different sample sizes, but together they suggest limited differentiation in the realized climatic niche during invasion. The overlapping climatic niche space was mainly associated with the monsoon climate zone from southern North China to the middle and lower reaches of the Yangtze River, consistent with the geographic co‐suitability patterns (Figures 4 and 8).

## 4 Discussion

### 4.1 Spatial Patterns of Suitable Habitat and Niche Overlap Under Current and Future Climate Scenarios

This study combined multiple SDM algorithms to project potential climatic suitability and overlap of *H. cunea* and its parasitoid *C. cunea* in China under the current climate baseline and future scenarios. Under the current baseline, the overlapping suitable area was 0.89 million km^2^, representing 54.46% of the suitable area for *H. cunea* (Table 2; Figure 4). The climatic niche overlap index Schoener's *D* was 0.738 (Figure 8a), indicating substantial overlap in climatic space (Broennimann et al. 2012). Co‐suitability was concentrated in the North China Plain, the middle and lower reaches of the Yangtze River Basin, and the Sichuan Basin, which are also regions with frequent *H. cunea* outbreaks.

Across future scenarios, both species were projected to show a general northward shift with modest changes in total suitable area (Figures 5 and 6). Under SSP5‐8.5 in the 2050s, suitable habitat for *H. cunea* was projected to increase to 1.91 million km^2^ and the overlapping suitable area with *C. cunea* to 1.15 million km^2^ (Tables 1 and 2; Figure 7), mainly through expansion towards North and Northeast China and some higher‐elevation regions. Nevertheless, overlap remained incomplete in several southern areas where *H. cunea* suitability persists but *C. cunea* suitability is low (Figures 4 and 7).

Climate warming can disrupt host‐parasitoid interactions through both spatial and temporal pathways. Spatially, different rates and directions of range shifts, dispersal limitation and habitat barriers can create geographic mismatches even when climatic niches overlap (Heikkinen et al. 2007; Walther et al. 2009). Temporally, warming can alter development rates, voltinism and diapause, potentially desynchronizing host availability and parasitoid emergence (Stireman III et al. 2005). Mismatch is more likely when climate change relaxes constraints for the host (e.g., warmer winters) but not for the parasitoid (e.g., narrower thermal tolerance, humidity dependence, or different diapause cues). Because biological control depends on close spatiotemporal matching, our maps identify where climatic co‐suitability is likely to persist and where mismatch may increase, but climate alone cannot capture all ecological constraints.

Importantly, incomplete overlap does not, by itself, demonstrate that natural enemies “lag” behind invasive hosts. Areas of predicted *H. cunea* suitability without corresponding *C. cunea* suitability already occur under the current climate baseline, particularly in parts of southern China, and this pattern is projected to persist under future scenarios (Figure 7). Such mismatch could arise from multiple non‐climatic factors, including host plant distribution, landscape connectivity, management history, and the fact that *C. cunea* occurrence records may reflect both natural populations and augmentative releases. Therefore, release or breeding programmes should be guided by both climatic suitability and local ecological feasibility; regions with high host suitability but low parasitoid suitability should be prioritized for monitoring and for evaluating complementary control options rather than assuming that releases will automatically establish.

### 4.2 Differences Between the Ecological Niche Responses of the Two Insects to Climate Variables and Human Disturbance

Permutation‐based variable importance suggests that *H. cunea* suitability is most strongly associated with dry‐season moisture and winter cold constraints (bio14 and bio6), together with human influence (HII) (Table 4). This pattern is consistent with the ability of the host to persist across a broad climatic range while being limited by climatic extremes (Peterson 2003). For *C. cunea*, the highest importance was for HII, followed by bio6 and precipitation seasonality (bio15) (Table 4). The strong association with HII may reflect the concentration of parasitoid records in human‐managed landscapes and/or the influence of mass‐rearing and release programmes; accordingly, interpretations should be cautious and should not be taken as evidence of a purely ecological preference.

Human activities may also shape the realized spatial match between host and parasitoid. In less accessible or low‐management regions (e.g., plateau margins and mountainous transition zones), *H. cunea* may establish through natural dispersal, whereas *C. cunea* establishment may be constrained by dispersal, habitat fragmentation, or insufficient release effort. Such context‐dependence can create “control gaps” where host presence is not matched by effective parasitoid pressure (Paini et al. 2016). Combining climatic suitability with landscape and management information, and evaluating establishment success through field monitoring, will help improve risk assessment and refine spatially targeted biological control (Van Lenteren 2012).

## 5 Conclusion

This study evaluated climatic suitability and potential spatial overlap between *H. cunea* and its parasitoid *C. cunea* under current and future climate scenarios. We found that (1) under the current climate baseline, co‐suitable habitat covers approximately 0.89 million km^2^ (54.46% of the suitable area for *H. cunea* ), indicating substantial potential for spatial co‐occurrence; (2) projections suggest a general northward shift for both species, with North and Northeast China remaining key regions of potential co‐suitability under several scenarios; (3) climatic niche overlap is substantial (Schoener's *D* = 0.738), but co‐suitability remains incomplete and may be shaped by non‐climatic constraints and management; and (4) under SSP5‐8.5, the overlapping suitable area could increase to approximately 1.15 million km^2^ by the 2050s, highlighting regions where monitoring and evaluation of biocontrol feasibility may be prioritized. In summary, addressing biological invasions under climate change requires integrating climatic suitability with ecological constraints and management context to improve the spatial matching of hosts and their natural enemies. The modeling framework and analytical approach used here can support risk monitoring and can be adapted to investigate linked responses of other invasive species and their natural enemies.

## Author Contributions

**Xianheng Ouyang:** conceptualization (lead), data curation (lead), investigation (lead), methodology (lead), software (lead), visualization (lead), writing – original draft (lead), writing – review and editing (lead). **Xiaoning Nan:** investigation (lead), methodology (lead), visualization (lead), writing – review and editing (lead). **Fushi Zhong:** methodology (lead), software (lead). **Qiaoyun Sun:** funding acquisition (equal), project administration (equal), writing – review and editing (equal). **Yang Liu:** conceptualization (equal), project administration (equal), supervision (equal), validation (equal), writing – review and editing (equal).

## Funding

This work was supported by Start‐up funds Research for Northwest A&F University, Z1090124092, National Natural Science Foundation of China (No. 32501717). GuangDong Basic and Applied Basic Research Foundation, 2023A1515110856.

## Conflicts of Interest

The authors declare no conflicts of interest.

## Supporting information


**Figure S1:** Changes in the habitat suitable for *Hyphantria cunea* under different future climate change scenarios.
Note: SSP1‐2.6, SSP2‐4.5, and SSP5‐8.5: low, medium, and high emission scenarios, respectively.
**Figure S2:** Future possible changes in the overlapping habitats suitable for *Hyphantria cunea* and its parasitoid, *Chouioia cunea* under different future climate change scenarios.
Note: Change categories were derived by comparing binary suitable areas (above MTSS) between the current baseline and each future projection. Decrease (contraction) indicates areas suitable currently but unsuitable in the future; Constant (stability) indicates areas suitable in both periods; Expansion indicates newly suitable areas in the future. SSP1‐2.6, SSP2‐4.5 and SSP5‐8.5 represent low, intermediate and high emission pathways, respectively. 2030s and 2050s correspond to 2021–2040 and 2041–2060, respectively.

**Table S1:** Environmental variables.
**Table S2:** Predictor screening, collinearity control and ecological rationale.
**Table S3:** Spatial variation in potential geographical distribution area (×10^4^ km^2^) for *Hyphantria cunea* under future climate scenarios.

## Data Availability

Occurrence records used in this study are publicly available from GBIF (https://www.gbif.org) and BOLD (https://www.boldsystems.org), and environmental predictors were downloaded from WorldClim v2.1 (https://www.worldclim.org/) and NASA SEDAC (Human Influence Index). All the required data are uploaded as Supporting Information.
