# Supplementary material for: Climate Change Shapes Suitable Habitat and Ecological Niche Overlap Between Hyphantria cunea and Its Parasitoid Chouioia cunea in China
Source: Ecol Evol. 2026 Apr 28;16(5):e73469. doi: 10.1002/ece3.73469 (PMC13124676; doi:10.1002/ece3.73469)
Supplement: Supplementary file 1 — Figure S1: Changes in the habitat suitable for Hyphantria cunea under different future climate change scenarios. Note: SSP1‐2.6, SSP2‐4.5, and SSP5‐8.5: low, medium, and high emission scenarios, respectively. Figure S2: Future possible changes in the overlapping habitats suitable for Hyphantria cunea and its parasitoid, Chouioia cunea under different future climate change scenarios. Note: Change categories were derived by comparing binary suitable areas (above MTSS) between the current baseline and each future projection. Decrease (contraction) indicates areas suitable currently but unsuitable in the future; Constant (stability) indicates areas suitable in both periods; Expansion indicates newly suitable areas in the future. SSP1‐2.6, SSP2‐4.5 and SSP5‐8.5 represent low, intermediate and high emission pathways, respectively. 2030s and 2050s correspond to 2021–2040 and 2041–2060, respectively. [file ECE3-16-e73469-s002.zip › FigS1-FigS2/FigS1-FigS2.docx]

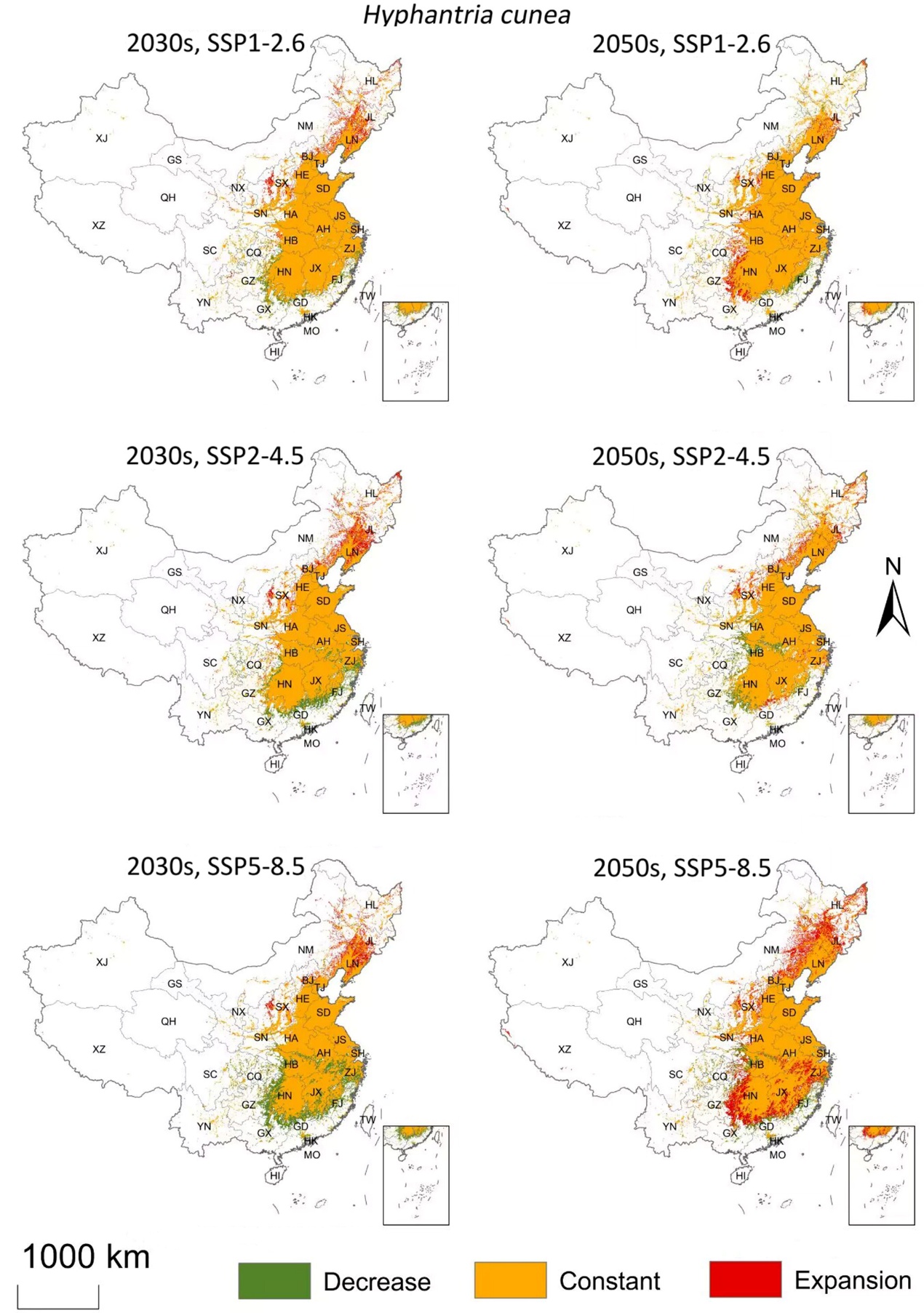


Figure S1. Changes in the habitat suitable for Hyphantria cunea under different future climate change scenarios.

Note: SSP1-2.6, SSP2-4.5, and SSP5-8.5: low, medium, and high emission scenarios, respectively.


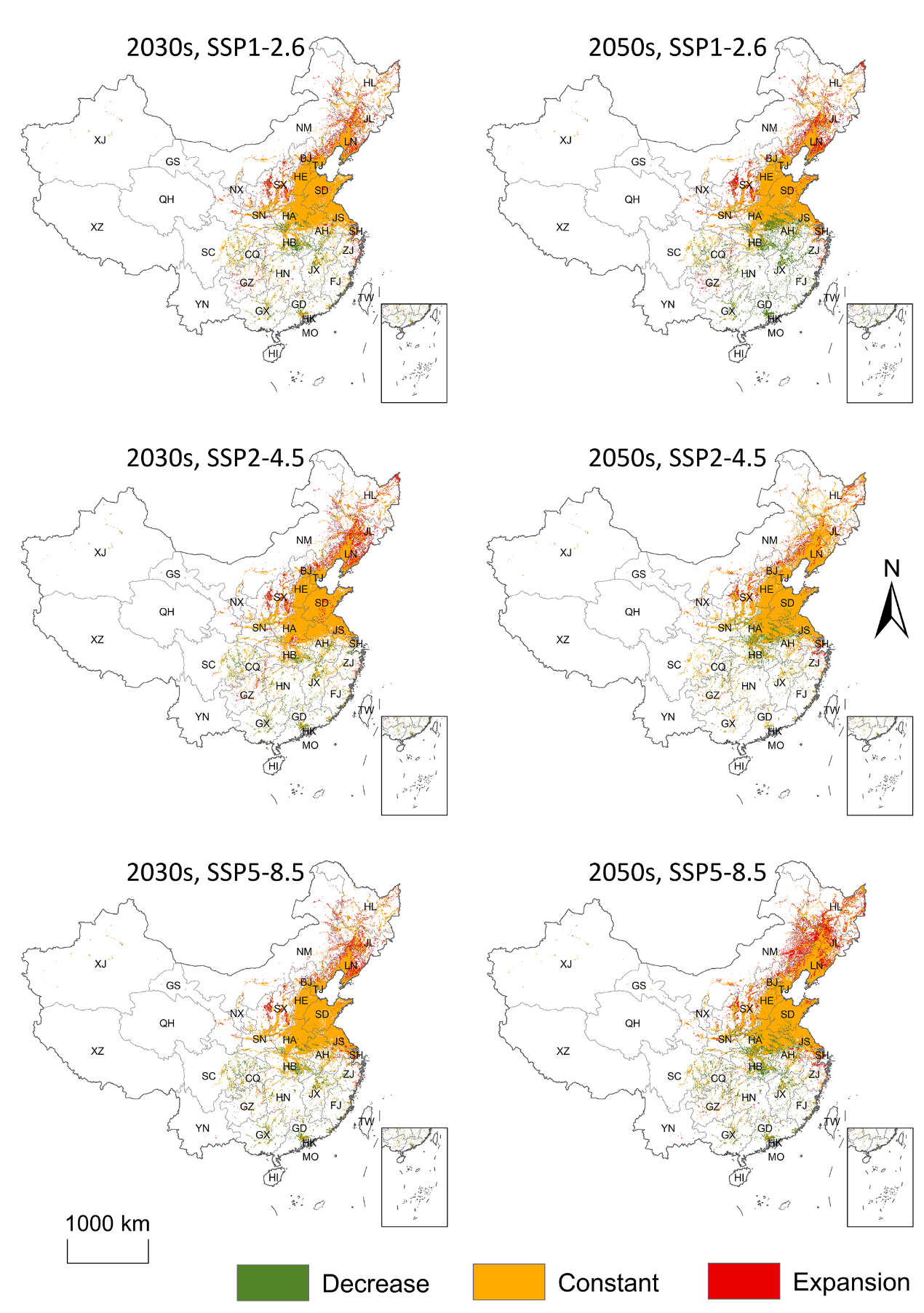


Figure S2. Future possible changes in the overlapping habitats suitable for *Hyphantria cunea* and its parasitoid, *Chouioia cunea* under different future climate change scenarios.

Note: Change categories were derived by comparing binary suitable areas (above MTSS) between the current baseline and each future projection. Decrease (contraction) indicates areas suitable currently but unsuitable in the future; Constant (stability) indicates areas suitable in both periods; Expansion indicates newly suitable areas in the future. SSP1-2.6, SSP2-4.5 and SSP5-8.5 represent low, intermediate and high emission pathways, respectively. 2030s and 2050s correspond to 2021-2040 and 2041-2060, respectively.
