# Supplementary material for: Climate Change Shapes Suitable Habitat and Ecological Niche Overlap Between Hyphantria cunea and Its Parasitoid Chouioia cunea in China
Source: Ecol Evol. 2026 Apr 28;16(5):e73469. doi: 10.1002/ece3.73469 (PMC13124676; doi:10.1002/ece3.73469)
Supplement: Supplementary file 2 — Table S1: Environmental variables. Table S2: Predictor screening, collinearity control and ecological rationale. Table S3: Spatial variation in potential geographical distribution area (×104 km2) for Hyphantria cunea under future climate scenarios. [file ECE3-16-e73469-s001.docx]

Supplementary table S1 Environmental variables Variables.

| Variables | Description | Unit | Resolution | Sources |
| --- | --- | --- | --- | --- |
| bio1 | Annual mean temperature | ℃ | 2.5 arc minutes | WorldClim |
| bio2 | Mean diurnal range | ℃ | 2.5 arc minutes | WorldClim |
| bio3 | Isothermality | - | 2.5 arc minutes | WorldClim |
| bio4 | Temperature seasonality | ℃ | 2.5 arc minutes | WorldClim |
| bio5 | Max temperature of warmest month | ℃ | 2.5 arc minutes | WorldClim |
| bio6 | Min temperature of coldest month | ℃ | 2.5 arc minutes | WorldClim |
| bio7 | Temperature annual range | ℃ | 2.5 arc minutes | WorldClim |
| bio8 | Mean temperature of wettest quarter | ℃ | 2.5 arc minutes | WorldClim |
| bio9 | Mean temperature of driest quarter | ℃ | 2.5 arc minutes | WorldClim |
| bio10 | Mean temperature of warmest quarter | ℃ | 2.5 arc minutes | WorldClim |
| bio11 | Mean temperature of coldest quarter | ℃ | 2.5 arc minutes | WorldClim |
| bio12 | Annual precipitation | mm | 2.5 arc minutes | WorldClim |
| bio13 | Precipitation of wettest month | mm | 2.5 arc minutes | WorldClim |
| bio14 | Precipitation of driest month | mm | 2.5 arc minutes | WorldClim |
| bio15 | Precipitation seasonality | - | 2.5 arc minutes | WorldClim |
| bio16 | Precipitation of wettest quarter | mm | 2.5 arc minutes | WorldClim |
| bio17 | Precipitation of driest quarter | mm | 2.5 arc minutes | WorldClim |
| bio18 | Precipitation of warmest quarter | mm | 2.5 arc minutes | WorldClim |
| bio19 | Precipitation of coldest quarter | mm | 2.5 arc minutes | WorldClim |
| altitude | Altitude | m | 2.5 arc minutes | WorldClim |
| hii | Human Influence Index | - | 2.5 arc minutes | NASA Socioeconomic Data and Applications Center |

Table S2. Predictor screening, collinearity control and ecological rationale.

| Variable | Description and selection rationale |
| --- | --- |
| bio1 | Annual mean temperature. Excluded — removed during multicollinearity screening (\|r\| > 0.8) and considered redundant with retained temperature metrics (e.g., bio6, bio7). |
| bio2 | Mean diurnal range. Excluded — removed during multicollinearity screening (\|r\| > 0.8) and considered redundant with retained temperature metrics (e.g., bio6, bio7). |
| bio3 | Isothermality. Excluded — removed during multicollinearity screening (\|r\| > 0.8) and considered redundant with retained temperature metrics (e.g., bio6, bio7). |
| bio4 | Temperature seasonality. Excluded — removed during multicollinearity screening (\|r\| > 0.8) and considered redundant with retained temperature metrics (e.g., bio6, bio7). |
| bio5 | Max temperature of warmest month. Excluded — removed during multicollinearity screening (\|r\| > 0.8) and considered redundant with retained temperature metrics (e.g., bio6, bio7). |
| bio6 | Min temperature of coldest month. Included (final predictor) — extreme winter cold relevant to overwinter survival. |
| bio7 | Temperature annual range. Included (final predictor) — captures seasonal thermal amplitude affecting development. |
| bio8 | Mean temperature of wettest quarter. Excluded — removed during multicollinearity screening (\|r\| > 0.8) and considered redundant with retained temperature metrics (e.g., bio6, bio7). |
| bio9 | Mean temperature of driest quarter. Excluded — removed during multicollinearity screening (\|r\| > 0.8) and considered redundant with retained temperature metrics (e.g., bio6, bio7). |
| bio10 | Mean temperature of warmest quarter. Excluded — removed during multicollinearity screening (\|r\| > 0.8) and considered redundant with retained temperature metrics (e.g., bio6, bio7). |
| bio11 | Mean temperature of coldest quarter. Excluded — removed during multicollinearity screening (\|r\| > 0.8) and considered redundant with retained temperature metrics (e.g., bio6, bio7). |
| bio12 | Annual precipitation. Excluded — removed during multicollinearity screening (\|r\| > 0.8) and considered redundant with retained precipitation metrics (e.g., bio14, bio15, bio18, bio19). |
| bio13 | Precipitation of wettest month. Excluded — removed during multicollinearity screening (\|r\| > 0.8) and considered redundant with retained precipitation metrics (e.g., bio14, bio15, bio18, bio19). |
| bio14 | Precipitation of driest month. Included (final predictor) — dry‑season moisture constraint relevant to host plants and larvae. |
| bio15 | Precipitation seasonality. Included (final predictor) — reflects intra‑annual variability in moisture regime. |
| bio16 | Precipitation of wettest quarter. Excluded — removed during multicollinearity screening (\|r\| > 0.8) and considered redundant with retained precipitation metrics (e.g., bio14, bio15, bio18, bio19). |
| bio17 | Precipitation of driest quarter. Excluded — removed during multicollinearity screening (\|r\| > 0.8) and considered redundant with retained precipitation metrics (e.g., bio14, bio15, bio18, bio19). |
| bio18 | Precipitation of warmest quarter. Included (final predictor) — moisture during warm season linked to larval feeding period. |
| bio19 | Precipitation of coldest quarter. Included (final predictor) — moisture during cold season linked to overwintering conditions. |
| altitude | Altitude. Included (final predictor) — proxy for topographic and temperature gradients. |
| hii | Human Influence Index. Included (final predictor) — captures anthropogenic pressure and management/sampling context. |

Table S3. Spatial variation in potential geographical distribution area (×10^4^ km^2^) for *Hyphantria cunea* under future climate scenarios.

| Period | Scenario | Degree of habitat suitability (×10^4^ km^2^) | | |
| --- | --- | --- | --- | --- |
|  |  | Contraction | Stability | Expansion |
| 2030s | SSP1-2.6 | 8.48 | 155.02 | 15.27 |
|  | SSP2-4.5 | 14.38 | 149.11 | 17.13 |
|  | SSP5-8.5 | 26.40 | 137.10 | 14.73 |
| 2050s | SSP1-2.6 | 4.76 | 165.53 | 11.16 |
|  | SSP2-4.5 | 11.61 | 154.64 | 12.09 |
|  | SSP5-8.5 | 1.03 | 150.80 | 40.34 |
